# Evaluation of the Possible Anticonvulsant Effect of Δ^9^-Tetrahydrocannabinolic Acid in Murine Seizure Models

**DOI:** 10.1089/can.2020.0073

**Published:** 2022-02-10

**Authors:** Melissa J. Benson, Lyndsey L. Anderson, Ivan K. Low, Jia Lin Luo, Richard C. Kevin, Cilla Zhou, Iain S. McGregor, Jonathon C. Arnold

**Affiliations:** ^1^ Brain and Mind Centre, The University of Sydney, Sydney, New South Wales, Australia.; ^2^ Lambert Initiative for Cannabinoid Therapeutics, The University of Sydney, Sydney, New South Wales, Australia.; ^3^ Faculty of Science, School of Psychology, The University of Sydney, Sydney, New South Wales, Australia.; ^4^ Discipline of Pharmacology, Faculty of Medicine and Health, School of Medical Sciences, The University of Sydney, Sydney, New South Wales, Australia.

**Keywords:** medicinal cannabis, epilepsy, THCA, Dravet syndrome, seizure

## Abstract

**Introduction:** The cannabinoid Δ^9^-tetrahydrocannabinolic acid (Δ^9^-THCA) has long been suggested in review articles and anecdotal reports to be anticonvulsant; yet, there is scant evidence supporting this notion. The objective of this study was to interrogate the anticonvulsant potential of Δ^9^-THCA in various seizure models—the *Scn1a*^+/−^ mouse model of Dravet syndrome, the 6-Hz model of psychomotor seizures and the maximal electroshock (MES) model of generalized tonic-clonic seizures.

**Materials and Methods:** We examined the effect of acute Δ^9^-THCA treatment against hyperthermia-induced seizures, and subchronic treatment on spontaneous seizures and survival in the *Scn1a*^+/−^ mice. We also studied the effect of acute Δ^9^-THCA treatment on the critical current thresholds in the 6-Hz and MES tests using outbred Swiss mice. Highly purified Δ^9^-THCA was used in the studies or a mixture of Δ^9^-THCA and Δ^9^-THC.

**Results:** We observed mixed anticonvulsant and proconvulsant effects of Δ^9^-THCA across the seizure models. Highly pure Δ^9^-THCA did not affect hyperthermia-induced seizures in *Scn1a*^+/−^ mice. A Δ^9^-THCA/Δ^9^-THC mixture was anticonvulsant in the 6-Hz threshold test, but purified Δ^9^-THCA and Δ^9^-THC had no effect. Conversely, both Δ^9^-THCA and Δ^9^-THC administered individually were proconvulsant in the MES threshold test but had no effect when administered as a Δ^9^-THCA/Δ^9^-THC mixture. The Δ^9^-THCA/Δ^9^-THC mixture, however, increased spontaneous seizure severity and increased mortality of *Scn1a*^+/−^ mice.

**Discussion:** The anticonvulsant profile of Δ^9^-THCA was variable depending on the seizure model used and presence of Δ^9^-THC. Because of the unstable nature of Δ^9^-THCA, further exploration of Δ^9^-THCA through formal anticonvulsant drug development is problematic without stabilization. Future studies may better focus on determining the mechanisms by which combined Δ^9^-THCA and Δ^9^-THC alters seizure thresholds, as this may uncover novel targets for the control of refractory partial seizures.

## Introduction

Epilepsy is a common neurological disease with a lifetime prevalence of 7.6 per 1000 persons.^1^ Approximately 30% of epilepsy patients are refractory to currently available treatments, motivating the quest for novel treatment options.^2^ In recent years, there has been increasing interest in cannabis-based medicines as a source of novel anticonvulsant agents. This follows numerous media stories illuminating remarkable improvements in intractable childhood epilepsy patients using cannabis-based products,^3,4^ as well as the cannabidiol (CBD) formulation Epidiolex™ being approved by the US Food and Drug Administration (FDA) and European Medicines Agency (EMA) for the treatment of Dravet syndrome and Lennox–Gastaut syndrome.^5–7^

Despite CBD gaining regulatory approval, many patients continue to use unregulated, artisanal cannabis-based products that contain a multitude of cannabinoids. Frequently, these artisanal extracts contain very low amounts of CBD, leading to speculation that constituents beyond CBD have anticonvulsant activity.^8^ Indeed, many believe that Δ^9^-tetrahydrocannabinolic acid (Δ^9^-THCA), the biosynthetic precursor of Δ^9^-tetrahydrocannabinol (Δ^9^-THC), mediates the anticonvulsant efficacy of these products.^8–10^ Community use of Δ^9^-THCA for epilepsy occurs despite scant evidence to support its anticonvulsant properties.

Clinical evaluation of Δ^9^-THCA as an anticonvulsant is limited to two published studies, a case series and an open-label retrospective chart review, both in pediatric populations.^9,10^ The case series reported conflicting reductions and exacerbations of seizure frequency in four patients after the addition of relatively low doses (0.02–2.2 mg/kg/day oral) of Δ^9^-THCA to existing anticonvulsant regimens.^9^ The chart review reported that Δ^9^-THCA was ineffective in five patients using Δ^9^-THCA-only extracts.^10^

Over 40 years ago, a preclinical study showed that 200 mg/kg Δ^9^-THCA was anticonvulsant in the mouse maximal electroshock (MES) test.^11^ Since this study, Δ^9^-THCA has been attributed anticonvulsant activity in several reviews and lay media; yet, the evidence to support these assertions has not advanced beyond this original preclinical report.^12–14^

In this study, we evaluated the anticonvulsant potential of Δ^9^-THCA in the *Scn1a*^+/−^ mouse model of Dravet syndrome. In addition, we examined its effects in two conventional seizure models: the 6-Hz threshold (6-HzT) model of psychomotor seizures and the MES threshold (MEST) model of generalized tonic-clonic seizures (GTCS).

## Materials and Methods

### Drugs

Δ^9^-THCA was isolated from hemp extracts. In brief, crude cannabis extract was dissolved in methanol (LiChrosolv^®^; Merck, Darmstadt, Germany) and treated overnight with activated charcoal (Ajax Finechem, Wollongong, Australia) at 4°C. The solution was filtered through a Büchner funnel and the filtrate was collected. The solvent was removed under pressure, and then reverse phase column chromatography (Büchi Reveleris PREP; Büchi AG, Flawil, Switzerland) with a C18 column (Büchi AG) was used to purify the residue and elute Δ^9^-THCA. Purity of Δ^9^-THCA isolated was 97% with 3% Δ^9^-THC. In addition, we purchased Δ^9^-THCA-A with a purity of 99.5% (<0.5% Δ^9^-THC content) and Δ^9^-THC (dronabinol, 100% purity) from THC Pharm GmbH (Frankfurt, Germany). Cannabinoids were stored protected from light at −30°C. Sodium valproate was purchased from Sigma-Aldrich, Inc. (St. Louis, MO). Analytical standards were purchased from Novachem Pty Ltd (Heidelberg West, Australia).

### Drug administration

Drug solutions were prepared fresh and were administered acutely as an intraperitoneal (*i.p*.) injection in a volume of 10 ml/kg. For conventional seizure model experiments (6-Hz and MEST), Δ^9^-THCA and Δ^9^-THC were prepared in 0.5% ethanol in vegetable oil. For hyperthermia-induced seizure experiments, Δ^9^-THCA was prepared in vegetable oil. Sodium valproate was prepared in saline.

### Purity analysis

Purity of Δ^9^-THCA was assessed by UV chromatography using Zorbax XDB-C18 column (Agilent Technologies, Inc., Santa Clara, CA) with a Shimadzu Nexera ultrahigh-performance liquid chromatograph coupled to a Shimadzu SPD-20AV photodiode array detector (Shimadzu Corp., Kyoto, Japan). Purity was calculated as a percent of the Δ^9^-THCA peak area to total peak area in the chromatogram at 272 nm (measured UV maxima of Δ^9^-THCA). Peak identity was confirmed by comparing retention time and UV spectra to a certified Δ^9^-THCA reference standard.

### Animals

All animal care and experimental procedures were approved by the University of Sydney Animal Ethics Committee in accordance with the Australian Code of Practice for the Care and Use of Animals for Scientific Purposes (2016/1035 and 2018/1395). Swiss outbred mice were purchased from Animal Resources Centre (stock ARC(S); Canning Vale, Australia) and singly housed after arrival for 7 days before experimentation. *Scn1a*^+/−^ mice were purchased from The Jackson Laboratory (stock 37107-JAX; Bar Harbor, ME) and generated for experiments as previously described.^15,16^ *Scn1a*^+/−^ mice were group housed. All mice were housed under a 12-h light/12-h dark cycle (07:00–19:00 light) with *ad libitum* access to food and water.

### Hyperthermia-induced seizures in *Scn1a*^+/−^ mice

Hyperthermia-induced seizure experiments were conducted on male and female *Scn1a*^+/−^ mice at postnatal days 14–16 (P14–16) as previously described.^15^ This model has been validated with first-line treatments, clobazam and valproic acid, and the phytocannabinoid CBD is anticonvulsant against hyperthermia-induced seizures in *Scn1a*^+/−^ mice.^17,18^ In brief, mice received a single *i.p.* injection of vehicle or Δ^9^-THCA by a researcher blinded to treatment and the hyperthermia protocol commenced immediately. Instantly following the hyperthermia-induced seizure protocol (duration ∼15 min), plasma and brains samples were collected and stored at −80°C until assayed.

### Spontaneous seizures and survival in *Scn1a*^+/−^ mice

Male and female *Scn1a*^+/−^ mice were exposed to a single hyperthermia-induced seizure event at P18 as described previously.^16^ Mice were randomly assigned to treatment groups after the thermally induced seizure. The mice were administered the cannabinoids orally through supplementation in chow. Δ^9^-THCA was dissolved in cold-pressed hemp seed oil (HempFoods Australia; Bangalow, Australia) and then formulated in R&M Standard Diet powder (Specialty Feeds; Glen Forrest, Australia). The final hemp seed oil concentration was 25% (v/w).

The groups tested were as follows: (1) control (hemp seed oil), (2) 250 mg Δ^9^-THCA/kg chow, and (3) 2000 mg Δ^9^-THCA/kg chow. An observer blinded to treatment quantified the number of spontaneous GTCS in a 60 h window.^15^ Mice continued drug treatment to P30 to monitor survival. Plasma and brain samples were collected on P31 within 30 min of lights on.

### MEST and 6-HzT tests

MEST and 6-HzT tests were conducted in Swiss male mice (9–12 weeks old) using a rodent electroconvulsive therapy (ECT) unit (Model 57800; Ugo Basile, Gemonio, Italy) as described previously.^19^ Mice were pretreated with vehicle, Δ^9^-THCA, Δ^9^-THC, or sodium valproate by *i.p.* injection 15 min before seizure induction. A 0.5% tetracaine in saline solution was applied to both corneas to induce local anesthesia. Pretreatment time (15 min) was based on previously determined time-to-peak plasma concentrations.^16,17^ Immediately before the electrical stimulation, saline was applied to each cornea to ensure electrical conductivity.

Corneal electroshocks (6 Hz, 3 s shock duration, 0.2 ms rectangular pulse width) starting at 20 mA and moving in 2 mA increments to a maximum of 50 mA were used for 6-HzT seizure experiments. Shocks were delivered and seizures were scored by an observer blinded to treatment for the presence of a psychomotor seizure occurring within 30 s of the shock delivery. Seizure response was characterized by the presence of rhythmic jaw, forelimb clonus, immobility, and/or Straub tail.^20^

For MEST-induced seizures a modified paradigm was used to adapt to the ECT unit.^21^ Corneal electroshocks (60 Hz, 0.4 s shock duration, 0.5 ms rectangular pulse width) were administered starting at 50 mA and moving in 2 mA increments to a maximum of 60 mA. Mice were shocked and scored by an observer blinded to treatment for the presence of GTCS with full hindlimb extension (hindlimbs at a 180° angle to the torso).

For both MEST and 6-HzT tests, the critical current (mA) at which 50% of mice seized (CC_50_) was determined using the “up-and-down” method described by Kimball et al.^22^

Separate cohorts of mice were used to collect plasma and brain samples to mimic the concentrations of Δ^9^-THCA and Δ^9^-THC at the time of 6-HzT seizure testing. Mice (*n*=6 per group) received an *i.p*. injection of Δ^9^-THCA (200 mg/kg, 97% purity) or Δ^9^-THC (6 mg/kg) and tetracaine was applied to corneas. Fifteen minutes later, saline was applied and mice received a standardized electroshock of 28 mA or 16 mA, the previously determined CC_50_ values for Δ^9^-THCA and Δ^9^-THC, respectively. Immediately after the electroshock, plasma and brain samples were collected through cardiac puncture.

### Analytical chemistry

Cannabinoid concentrations in biological samples were assayed by liquid chromatography–mass spectrometry (LC-MS)/MS as previously described.^17^ In brief, plasma samples were prepared using supported-liquid extraction (SLE) with methyl tert-butyl ether. Brain samples were prepared by filtering homogenates through Amicon Ultracel-3K (Merck-Millipore, Burlington, VT) filtration devices before SLE. Plasma and brain samples were reconstituted in acetonitrile and 0.1% formic acid in water (1:1, v/v) for analysis.

Samples were assayed by LC-MS/MS as previously described.^17,23^ The mass spectrometer operated in negative (Δ^9^-THCA) and positive (Δ^9^-THC) electrospray ionization modes with multiple reaction monitoring and the following mass transition pairs: *m/z* 357.20→245.35, 357.20→191.30 (Δ^9^-THCA) and *m/z* 315.15→193.15, 315.15→259.20 (Δ^9^-THC). Quantification was achieved by comparing experimental samples to 8-point standard curves prepared with analytical standards. Limits of quantification (LOQ) were 0.04 ng/mg brain and <50 ng/ml plasma (Δ^9^-THCA), 0.1 ng/ml plasma and 0.005 ng/mg brain (Δ^9^-THC).

### Statistical analysis

Hyperthermia-induced seizure threshold temperatures and survival data were analyzed using the Mantel–Cox log-rank test. Statistical comparisons of spontaneous seizure data were made using Fisher's exact test (proportion of mice seizure free) or one-way analysis of variance (ANOVA) followed by Dunnett's *post hoc* (seizure frequency and seizure severity). MEST and 6-HzT data were analyzed using one-way ANOVA followed by Dunnett's *post hoc* comparisons. Plasma Δ^9^-THC concentrations were analyzed using a Student's *t*-test. *p*<0.05 was considered statistically significant for all analyses.

## Results

### Purified Δ^9^-THCA is ineffective against hyperthermia-induced seizures in *Scn1a*^+/−^ mice

**FIG. 1.**
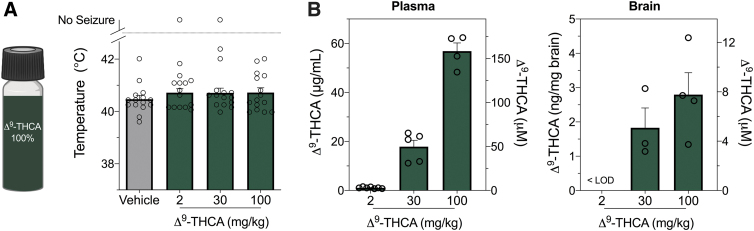
The effects of Δ^9^-THCA on hyperthermia-induced seizures in *Scn1a*^+/−^ mice. **(A)** Pure Δ^9^-THCA that contained <0.5% THC impurity was used for hyperthermia-induced seizure experiments in *Scn1a*^+/−^ mice. Threshold temperature of individual mice for GTCS induced by hyperthermia after acute *i.p.* treatment with vehicle (VEH, gray bar) or varying doses of pure Δ^9^-THCA (dark green bars). Δ^9^-THCA had no effect on the temperature threshold for hyperthermia-induced seizures. The average temperatures of seizure induction are depicted by the bars and error bars represent SEM, with *n*=15 per group (log-rank Mantel–Cox). **(B)** Concentrations of Δ^9^-THCA in plasma (left panel) and brain (right panel) from individual experimental animals. Concentrations are depicted as both mass concentrations (left *y*-axis) and molar concentrations (right *y*-axis). Error bars represent SEM, with *n*=4–7 per treatment. Δ^9^-THCA, Δ^9^-tetrahydrocannabinolic acid; GTCS, generalized tonic-clonic seizures; *i.p.*, intraperitoneal; LOD, limit of detection; SEM, standard error of the mean.

We evaluated pure Δ^9^-THCA against hyperthermia-induced seizures in the *Scn1a*^+/−^ mouse model of Dravet syndrome (Fig. 1A). Based on allometric scaling, a low Δ^9^-THCA dose (2 mg/kg) was administered to approximate low doses administered to childhood epilepsy patients.^8–10^ The highest dose tested (100 mg/kg) matched the dose of CBD that has been shown to be anticonvulsant against hyperthermia-induced seizures in *Scn1a*^+/−^ mice.^17,24^ No effect was observed on the temperature threshold for thermally induced seizures at any dose (Fig. 1A). Despite Δ^9^-THCA having a low brain-to-plasma ratio (<10%), micromolar concentrations were found in the brain at doses ≥30 mg/kg (Fig. 1B).

### Combined Δ^9^-THCA and Δ^9^-THC is anticonvulsant in the 6-HzT seizure model

**FIG. 2.**
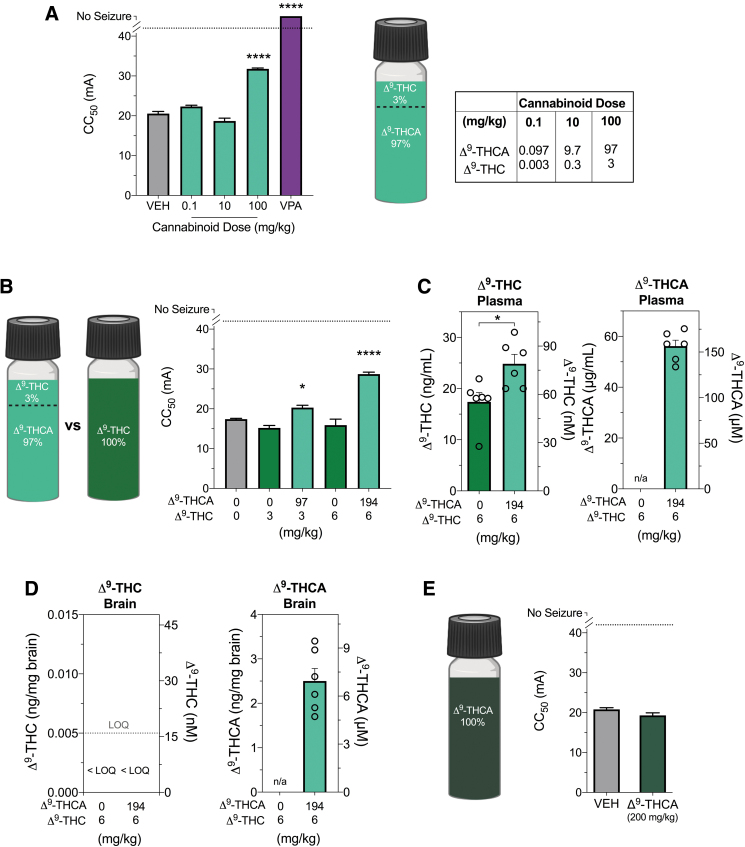
The effects of Δ^9^-THCA in the 6-HzT test. **(A)** The CC_50_ exhibit a psychomotor seizure in the 6-HzT seizure model after acute *i.p.* treatment with vehicle (VEH, gray bar), varying doses of a Δ^9^-THCA/Δ^9^-THC mixture (light green bars) or sodium valproate (VPA, purple bar). Δ^9^-THCA/Δ^9^-THC mixture (100 mg/kg) significantly increased the CC_50_ threshold. Sodium valproate (300 mg/kg) treatment protected mice from psychomotor seizures. Error bars represent SEM, with *n*=11–12 per treatment (****p<0.0001; one-way ANOVA followed by Dunnett's *post hoc* compared with vehicle-treated mice). Cannabinoid content of the Δ^9^-THCA/Δ^9^-THC mixture was 97% Δ^9^-THCA and 3% Δ^9^-THC, with the corresponding doses. **(B)** The 6-HzT test was repeated to compare CC_50_ values after treatment with Δ^9^-THCA/Δ^9^-THC mixture (light green bar) with those following treatment with matched doses of pure Δ^9^-THC (green bars). Δ^9^-THCA/Δ^9^-THC mixture (100 and 200 mg/kg) significantly increased the CC_50_, with *n*=12 per treatment (*p<0.05, ****p<0.0001; one-way ANOVA followed by Dunnett's *post hoc* compared with vehicle-treated mice). **(C)** Plasma and **(D)** brain concentrations of Δ^9^-THC (left panel) and Δ^9^-THCA (right panel) in individual animals after treatment with 6 mg/kg Δ^9^-THC (green bar) or 200 mg/kg Δ^9^-THCA/Δ^9^-THC formulation (light green bars). Significantly higher plasma Δ^9^-THC concentrations were observed after treatment with the Δ^9^-THCA/Δ^9^-THC mixture (*p<0.05, Student's *t*-test). Concentrations of Δ^9^-THC in brain samples were below the LOQ, depicted by the dashed line. Concentrations are depicted as both mass concentrations (left *y*-axis) and molar concentrations (right *y*-axis). Error bars represent SEM, with *n*=6 per treatment. **(E)** The CC_50_ value in the 6-HzT seizure model after acute *i.p.* treatment with vehicle (VEH, grey bar) or 200 mg/kg pure Δ^9^-THCA (dark green bar) that contained <0.5% Δ^9^-THC impurity. Error bars represent SEM, with *n*=12 per treatment (Student's *t*-test). ANOVA, analysis of variance; CC_50_, critical current at which 50% of mice seized; 6-HzT, 6-Hz threshold; LOQ, limit of quantification.

We then sought to examine the effects of Δ^9^-THCA (97% Δ^9^-THCA and 3% Δ^9^-THC) on psychomotor seizures using the 6-HzT test. Although initially the presence of Δ^9^-THC was undesirable, the effects of this mixture remains highly relevant to community usage of Δ^9^-THCA-dominant oils that contain both Δ^9^-THCA and Δ^9^-THC.^7^ This Δ^9^-THCA/Δ^9^-THC mixture was anticonvulsant in the 6-HzT seizure model (one-way ANOVA; *F*_4,4_=332.5, *p*<0.0001); the 100 mg/kg dose significantly increased the CC_50_ compared with vehicle-treated mice (*p*=0.0002) (Fig. 2A); however, the effect size was small compared with sodium valproate (300 mg/kg), which yielded 100% protection (Fig. 2A).

We then determined whether the anticonvulsant effect observed at 100 mg/kg was simply attributed to Δ^9^-THC and whether a higher dose of the Δ^9^-THCA/Δ^9^-THC mixture had a more robust anticonvulsant effect. We repeated the experiment with 100 and 200 mg/kg doses of the Δ^9^-THCA/Δ^9^-THC mixture and Δ^9^-THC alone (3 and 6 mg/kg) matching the Δ^9^-THC doses found in the mixture (Fig. 2B). Again the Δ^9^-THCA/Δ^9^-THC mixture was anticonvulsant (*F*_4,55_=45.64, *p*<0.0001). The CC_50_ values of the Δ^9^-THCA/Δ^9^-THC mixture were significantly greater than vehicle (100 mg/kg, *p*=0.0497 and 200 mg/kg, *p*<0.0001).

Neither dose of Δ^9^-THC had any effect, suggesting that Δ^9^-THC within the Δ^9^-THCA/Δ^9^-THC mixture was not responsible for the anticonvulsant effect. We compared plasma and brain concentrations of Δ^9^-THC and Δ^9^-THCA from mice treated with 200 mg/kg of the Δ^9^-THCA/Δ^9^-THC mixture with those treated with a matched Δ^9^-THC (6 mg/kg) dose (Fig. 2C, D). Of interest, the addition of Δ^9^-THCA increased plasma Δ^9^-THC concentrations, with higher Δ^9^-THC concentrations observed in Δ^9^-THCA/Δ^9^-THC mixture group than the matched Δ^9^-THC-alone group (*p*=0.0170). Brain Δ^9^-THC concentrations were detectable but below the LOQ (Fig. 2D). The brain Δ^9^-THC concentrations would have been low and rising 15 min postdose, as the brain *t*_max_ of Δ^9^-THC is 60–120 min.^25,26^ A mean Δ^9^-THCA concentration of 6.98 (±1.93) μM was measured in brain tissue (Fig. 2D).

Subsequently, we sourced pure Δ^9^-THCA (<0.5% THC impurity) to examine its effects in the 6-HzT test (Fig. 2E). Pure Δ^9^-THCA (200 mg/kg) had no effect on the threshold of seizures induced by 6-Hz electroshock.

### Δ^9^-THCA is chemically unstable under controlled storage conditions

**FIG. 3.**
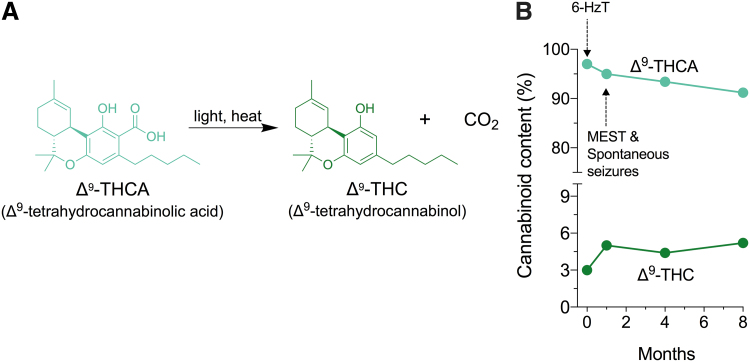
Δ^9^-THCA is chemically unstable. **(A)** Chemical structure of Δ^9^-THCA and schematic of its decarboxylation to Δ^9^-THC. Decarboxylation of Δ^9^-THCA is catalyzed by light and heat. **(B)** Cannabinoid content of the Δ^9^-THCA-dominant cannabinoid extract over time. Δ^9^-THCA was stored protected from light at −30°C. Arrows represent when experiments were conducted.

When exposed to light and/or heat, Δ^9^-THCA readily decarboxylates to Δ^9^-THC (Fig. 3A). The Δ^9^-THCA-dominant mixture was stored protected from light at −30°C and cannabinoid content was assessed over time (Fig. 3B). Despite these storage conditions, Δ^9^-THCA was not stable and degraded to 91% over 8 months explaining the different Δ^9^-THCA to Δ^9^-THC ratios across our experiments.

### Purified Δ^9^-THCA and Δ^9^-THC administered alone are proconvulsant in the MEST seizure model

**FIG. 4.**
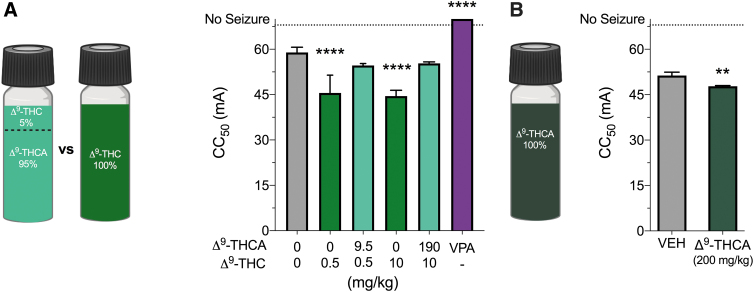
The effects of Δ^9^-THCA in the MEST test. **(A)** A Δ^9^-THCA/Δ^9^-THC mixture and pure Δ^9^-THC were used in the MEST acute seizure model. Cannabinoid content of the Δ^9^-THCA/Δ^9^-THC mixture was 95% Δ^9^-THCA and 5% Δ^9^-THC. The CC_50_ exhibits a seizure with maximal hindlimb extension after acute *i.p.* treatment with vehicle (VEH, gray bar), pure Δ^9^-THC (green bar), a Δ^9^-THCA/Δ^9^-THC mixture (light green bar), or sodium valproate (VPA, purple bar). Dose of pure Δ^9^-THC matches that in the Δ^9^-THCA/Δ^9^-THC mixture. Δ^9^-THC (0.5 and 10 mg/kg) significantly reduced the CC_50_ threshold for MES seizures. Sodium valproate (300 mg/kg) treatment protected mice from MES-induced tonic extension. Error bars represent SEM, with *n*=12 per treatment (****p<0.0001; one-way ANOVA followed by Dunnett's *post hoc* compared with vehicle-treated mice). **(B)** The MEST test was repeated to compare CC_50_ values after treatment with vehicle (VEH, grey bar) or 200 mg/kg pure Δ^9^-THCA (dark green bar) that contained <0.5% Δ^9^-THC impurity. Δ^9^-THCA treatment significantly reduced the CC_50_ compared with vehicle treatment. Error bars represent SEM, with *n*=12 per treatment (**p<0.01; Student's *t*-test). MEST, maximal electroshock threshold.

We examined the effect of the Δ^9^-THCA/Δ^9^-THC mixture (95% Δ^9^-THCA, 5% Δ^9^-THC) in the MEST model of GTCS (Fig. 4A). We conducted a MEST test with a 200 mg/kg dose of the Δ^9^-THCA/Δ^9^-THC mixture and purified Δ^9^-THC at 10 mg/kg to match the dose in the mixture (Fig. 4A). To assess potential low-dose effects of Δ^9^-THCA and Δ^9^-THC, we also examined the effect of a lower dose of the Δ^9^-THCA/Δ^9^-THC mixture (10 mg/kg) and purified Δ^9^-THC (0.5 mg/kg).

Δ^9^-THC was proconvulsant in the MEST test (*F*_5,65_=52.55, *p*<0.0001). The CC_50_ values of both 0.5 and 10 mg/kg doses of Δ^9^-THC alone were significantly decreased compared with vehicle (*p*<0.0001 and *p*<0.0001, respectively). Neither dose of the Δ^9^-THCA/Δ^9^-THC mixture affected the CC_50_ in the MEST test. In contrast, sodium valproate (300 mg/kg) achieved 100% seizure protection (*p*<0.0001).

Subsequently, we procured a pure Δ^9^-THCA formulation and examined its effects in the MEST test (Fig. 4B). Of interest, Δ^9^-THCA (200 mg/kg) was proconvulsant with a CC_50_ significantly lower than in vehicle-treated mice (*p*=0.0054).

### Combined Δ^9^-THCA and Δ^9^-THC increased the severity of spontaneous seizures and reduced the lifespan of *Scn1a*^+/−^ mice

**FIG. 5.**
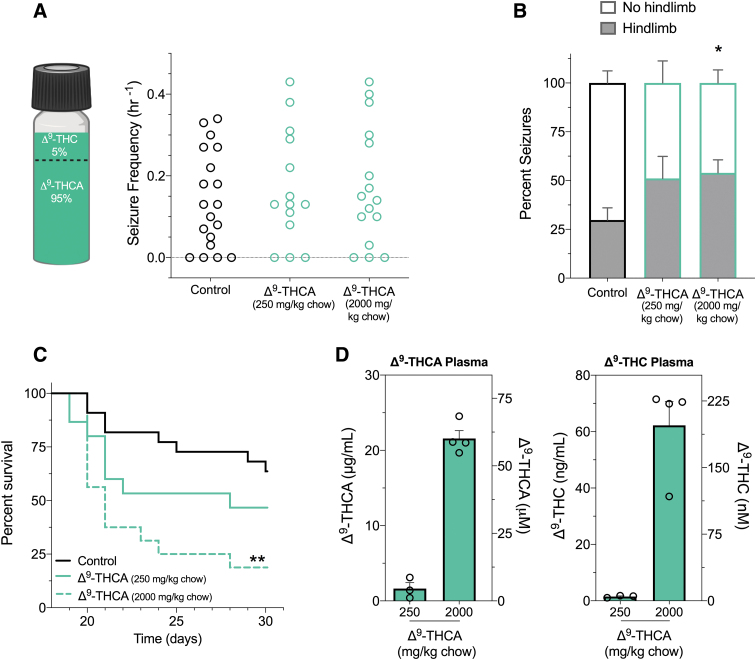
The effects of Δ^9^-THCA on spontaneous seizures and survival in *Scn1a*^+/−^ mice. **(A)** A Δ^9^-THCA/Δ^9^-THC mixture was used for spontaneous seizure and survival experiments in *Scn1a*^+/−^ mice. Cannabinoid content of the Δ^9^-THCA/Δ^9^-THC mixture used was 95% Δ^9^-THCA and 5% Δ^9^-THC. GTCS frequency of individual untreated and Δ^9^-THCA-treated mice is given. Treatments were administered orally through supplementation in chow, which was initiated after the induction of a single thermally induced seizure. Unprovoked, spontaneous GTCS were quantified over a 60-h recording period. Treatment with the Δ^9^-THCA/Δ^9^-THC mixture had no effect on incidence or frequency of seizures, with *n*=14–19 per group (Fisher's exact text and one-way ANOVA followed by Bonferroni's *post hoc*, respectively). **(B)** Proportion of spontaneous GTCS with (gray bars) or without (white bars) full tonic hindlimb extension is given. Subchronic treatment with high-dose Δ^9^-THCA/Δ^9^-THC mixture (2000 mg/kg chow) significantly increased the severity of GTCS in *Scn1a*^+/−^ mice. The proportion of GTCS with tonic hindlimb extension was significantly greater compared with control-treated mice (*p<0.05; Bonferroni's planned comparisons). Error bars represent SEM with *n*=11–15. **(C)** Survival curves comparing control and Δ^9^-THCA/Δ^9^-THC mixture-treated mice are given. Treatment began at postnatal day 18 (P18) and survival was monitored until P30. Survival of *Scn1a*^+/−^ mice was significantly worse with high-dose Δ^9^-THCA/Δ^9^-THC mixture (2000 mg/kg chow), with *n*=15–22 per group (**p<0.005; log-rank Mantel–Cox). **(D)** Plasma concentrations of Δ^9^-THCA (left panel) and Δ^9^-THC (right panel) from individual experimental animals treated with a Δ^9^-THCA/Δ^9^-THC mixture. Concentrations are depicted as both mass concentrations (left *y*-axis) and molar concentrations (right *y*-axis). Error bars represent SEM, with *n*=3–4 per treatment.

We then evaluated the effect of the Δ^9^-THCA/Δ^9^-THC mixture (95% Δ^9^-THCA, 5% Δ^9^-THC) against spontaneous seizures in *Scn1a*^+/−^ mice (Fig. 5A). Because these experiments require subchronic drug administration, it was not possible to procure sufficient quantities of purified Δ^9^-THCA. Treatment delivered through supplementation in chow (250 or 2000 mg/kg chow) had no effect on the proportion of mice that experienced spontaneous seizures or spontaneous seizure frequency (Fig. 5A). Treatment with Δ^9^-THCA/Δ^9^-THC mixture (2000 mg/kg chow) increased the severity of spontaneous seizures, as the percentage of seizures that advanced to hindlimb extension was significantly higher than control-treated mice (*p*=0.0139) (Fig. 5B).

Increased seizure severity was associated with poor survival, with only 19% survival to P30 compared with 64% survival of controls (*p*=0.0012) (Fig. 5C). Treatment with a lower dose of the Δ^9^-THCA/Δ^9^-THC mixture (250 mg/kg chow) had no effect on survival compared with controls. Steady-state plasma concentrations of Δ^9^-THCA and Δ^9^-THC measured in *Scn1a*^+/−^ experimental mice after subchronic treatment are given in Figure 5D. Concentrations of Δ^9^-THCA in the brain were below the LOQ for mice treated with the 250 mg/kg chow and 0.3±0.1 ng/mg brain (873±278 nM) with the 2000 mg/kg chow doses. Concentrations of Δ^9^-THC in brain samples were below the limit of detection and LOQ, respectively.

## Discussion

Δ^9^-THCA-dominant cannabis extracts are being used in the community to treat epilepsy despite insufficient evidence. We aimed to fill the knowledge gap by assessing the anticonvulsant properties of Δ^9^-THCA across several mouse seizure models. Our results highlight great complexity in the action of Δ^9^-THCA, with both anticonvulsant and proconvulsant effects being observed depending on the seizure model and presence of Δ^9^-THC. Against 6-Hz-induced seizures, Δ^9^-THCA was anticonvulsant only when Δ^9^-THC was present. However, in the MEST model, the Δ^9^-THCA/Δ^9^-THC mixture was ineffective and even proconvulsant when purified Δ^9^-THCA or Δ^9^-THC was administered alone. Finally, purified Δ^9^-THCA had no effect on hyperthermia-induced seizures in the *Scn1a*^+/−^ mouse model, whereas a Δ^9^-THCA/Δ^9^-THC mixture worsened spontaneous seizure severity and reduced survival.

This study further highlights the difficulties posed by the instability of Δ^9^-THCA for pharmacological research. Stability studies show that Δ^9^-THCA decarboxylates even when stored at 4 and 18°C, so Δ^9^-THC contamination in Δ^9^-THCA is “nearly unavoidable.”^27,28^ We observed significant decarboxylation of Δ^9^-THCA under conditions where it was stored protected from light at −30°C, with short exposures to air and ambient temperatures for drug preparation. Investigators characterizing the pharmacology of Δ^9^-THCA should be cognizant of its handling and storage conditions and routinely perform analytical tests to confirm purity.

In addition, those considering use of Δ^9^-THCA as a single molecule for pharmaceutical development might consider strategies to improve stability such as bioisosteric replacement of the carboxylic acid group.^29^ Although the instability of Δ^9^-THCA would need to be resolved before a formal drug development pathway, its degradation to Δ^9^-THC was not necessarily a disadvantage here. Understanding the effects of coadministered Δ^9^-THCA and Δ^9^-THC is highly relevant for epilepsy patients using Δ^9^-THCA-dominant cannabis extracts that invariably contain both cannabinoids, often with greater relative doses of Δ^9^-THCA to Δ^9^-THC.^8^

This study provides novel evidence that a Δ^9^-THCA/Δ^9^-THC mixture dose dependently reduced seizures in the 6-HzT test, although with mild effect sizes at very high doses (>100 mg/kg *i.p.*). Of importance, purified Δ^9^-THCA or Δ^9^-THC was ineffective when administered alone, which suggests a potential synergistic interaction between the two cannabinoids when combined. However, it is important to note that an isobolographic study would need to be conducted to draw a firm conclusion on the presence of cannabinoid synergy.

The current data are insufficient to draw such a conclusion. The interaction between Δ^9^-THCA and Δ^9^-THC might have pharmacodynamic and/or pharmacokinetic explanations. Because both cannabinoids were present in the brain, there could be a pharmacodynamic interaction at a common anticonvulsant target such as cannabinoid CB_1_ receptors. Recently, Δ^9^-THCA was reported to be a positive allosteric modulator of CB_1_ receptors.^30^ Alternatively, our observation that Δ^9^-THCA increased plasma concentrations of Δ^9^-THC points to a pharmacokinetic interaction that could be explored in future studies.

Although the Δ^9^-THCA/Δ^9^-THC mixture was effective in the 6-HzT model, it had no effect in the MEST test, and highly purified Δ^9^-THCA (200 mg/kg) was proconvulsant. This conflicts with a previous report showing that 200 mg/kg Δ^9^-THCA was anticonvulsant in the MES model.^11^ Unfortunately, this early study did not describe the purity of the Δ^9^-THCA that was used. Considerable Δ^9^-THC contamination might account for the effect because Δ^9^-THC is anticonvulsant in this model (Effective dose for 50% of cohort=35–43.8 mg/kg dose range).^31,32^ We found Δ^9^-THC to be proconvulsant at lower doses (0.5 and 10 mg/kg). This is consistent with a study reporting biphasic effects of Δ^9^-THC on the severity of MES seizures, with low doses having proconvulsant effects and high doses being anticonvulsant.^33^

Within the community, Δ^9^-THCA-dominant cannabis extracts are being used to treat Dravet syndrome patients even in the absence of evidence supporting its efficacy.^8,9^ In this study, highly purified Δ^9^-THCA had no effect on hyperthermia-induced seizures in the *Scn1a*^+/−^ mouse model of Dravet syndrome despite Δ^9^-THCA attaining >1 μM brain concentrations.

This is the first report of appreciable Δ^9^-THCA concentrations in brain tissue after systemic administration. However, it is important to clarify that Δ^9^-THCA does not readily accumulate in brain tissue as it has a low brain-to-plasma ratio (Fig. 1B, <10%). Our study's lowest dose corresponds to the highest dose reported by Sulak et al.^9^ in a case series of pediatric patients. It is possible that lower doses of Δ^9^-THCA might be effective given we have observed low-dose effects of Δ^9^-THC (0.1–0.3 mg/kg) against hyperthermia-induced seizures in *Scn1a*^+/−^ mice. Furthermore, very low doses of Δ^9^-THCA have been reported to reduce nausea and vomiting in rodents.^22,34^ The effects of lower doses of Δ^9^-THCA could be explored in a future study.

We also examined the effect of the Δ^9^-THCA/Δ^9^-THC mixture on spontaneous seizures and lifespan of *Scn1a*^+/−^ mice. This yielded catastrophic effects with the mixture worsening the severity of spontaneous seizures and reducing survival. A similar exacerbation of premature mortality was observed after cotreatment of CBD with Δ^9^-THC.^17^ A commonality between these studies is a pharmacokinetic interaction with the perpetrator drugs (CBD or Δ^9^-THCA) increasing the plasma concentrations of the victim drug (Δ^9^-THC). A recent study showed cannabinoid-induced convulsions may be a species-specific phenomenon that is restricted to rodents. However, the use of a high-dose Δ^9^-THCA-dominant extract was noted to exacerbate seizures in a Dravet syndrome patient, potentially refuting this possibility.^9,33^

The anticonvulsant efficacy of the Δ^9^-THCA/Δ^9^-THC mixture in the 6-HzT model warrants further exploration. Following the pathway of the NIH Epilepsy Therapy Screening Program, the Δ^9^-THCA/Δ^9^-THC mixture could be examined in the lamotrigine-resistant amygdala-kindled seizure model, which is used when an investigational drug is anticonvulsant in the 6-Hz but not the MES test. Levetiracetam, used to treat refractory partial seizures, is anticonvulsant in the 6-Hz but not the MES model.^20,35^ Therefore, it is conceivable that a Δ^9^-THCA/Δ^9^-THC mixture may have potential in treating therapy-resistant partial seizures, although the proconvulsant effects of purified Δ^9^-THCA in the MEST test and the THCA/Δ^9^-THC combination in *Scn1a*^+/−^ mice complicates its further development. In any case, Δ^9^-THCA is inferior to CBD as an anticonvulsant, with CBD displaying efficacy in the 6-Hz and MES seizure models, as well as the *Scn1a*^+/−^ mouse model of Dravet syndrome.^17,24,36–38^

## Conclusion

Our results suggest that Δ^9^-THCA-dominant medicinal cannabis formulations might be, at best, highly circumscribed in the treatment of epilepsy. Future studies may be better focused in determining the potential mechanisms by which Δ^9^-THCA alters seizure thresholds, as this may uncover novel targets for refractory seizure control.

## Abbreviations Used

Δ^9^-THCA: Δ^9^-tetrahydrocannabinolic acid
ANOVA: analysis of variance
ARC: Australian Research Council
CBD: cannabidiol
CC_50_: critical current at which 50% of mice seized
EMA: European Medicines Agency
FDA: US Food and Drug Administration
GTCS: generalized tonic-clonic seizures
6-HzT: 6-Hz threshold
*i.p.*: intraperitoneal
LOD: limit of detection
LOQ: limit of quantification
MEST: maximal electroshock threshold
NHMRC: National Health and Medical Research Council
SEM: standard error of the mean
VEH: vehicle
VPA: valproate
WHO: World Health Organization
